# Hepatoprotective Potential of Extracts from Seeds of *Areca catechu* and Nutgalls of *Quercus infectoria*

**DOI:** 10.3390/molecules14124987

**Published:** 2009-12-01

**Authors:** Pimolpan Pithayanukul, Saruth Nithitanakool, Rapepol Bavovada

**Affiliations:** 1 Department of Pharmacy, Faculty of Pharmacy, Mahidol University, Bangkok 10400, Thailand; E-Mail: saruth_pipe@yahoo.com (S.N.); 2 Department of Pharmaceutical Botany, Faculty of Pharmaceutical Sciences, Chulalongkorn University, Bangkok 10300, Thailand; E-Mail: brapepol2@hotmail.com (R.B.)

**Keywords:** anti-inflammatory, antioxidant, *Areca catechu* L., hepatoprotective activity, *Quercus infectoria* Oliv

## Abstract

Aqueous extracts from seeds of *Areca catechu* L. (Arecaceae) (AC) and nutgalls of *Quercus infectoria* Oliv. (Fagaceae) (QI) were investigated for their hepatoprotective potential by studying their antioxidant capacity using four different methods, by determining their *in vitro* anti−inflammatory activity against 5-lipoxygenase, and by evaluating their hepatoprotective potential against liver injury induced by carbon tetrachloride (CCl_4_) in rats. AC and QI extracts exhibited potent antioxidant and anti-inflammatory activities. Treatment of rats with AC and QI extracts reversed oxidative damage in hepatic tissues induced by CCl_4_. It is suggested that extracts rich in either condensed or hydrolysable tannins and known for their potent antioxidant and anti-inflammatory activities, may potentially confer protection against oxidative stress−induced liver injury. These data should contribute to evidence-based traditional medicines for anti-inflammatory and hepatoprotective effects of both extracts.

## 1. Introduction

Oxidative stress, a consequence of an imbalance of pro-oxidants and antioxidants in the organism, has been implicated in several disease states, including inflammation and liver disease [1]. Compounds that block or retard the chain reaction of oxidation are reported to prevent oxidative stress−induced hepatotoxicity [2]. A group of phenolic compounds known as tannins are significant plant secondary metabolites. Tannins occur in two types: condensed and hydrolysable tannins. These compounds are greatly varied in their structures and concentration within and among plant species. Various studies have demonstrated that tannins from plants exhibit hepatoprotective efficacy [3,4].

Areca nut (*Areca catechu* L., Arecaceae) (AC) is one of popular traditional herbal medicines used in Thailand and widely cultivated throughout Thailand and South Asia. The activities of areca seed are anthelmintic, antifungal, antibacterial, anti−inflammatory, antioxidant, insecticide and lavicidal [5]. In traditional Chinese medicine, areca seeds have been used in various formulations to treat liver disorders [6]. The polyphenolic constituents of its seed are chiefly condensed tannins of procyanidin dimers, trimers and tetramers [7].

In Asian countries, the galls of *Quercus infectoria* Oliv. (Fagaceae) (QI) have been used for centuries in traditional medicines for treating inflammatory diseases. Gargles of a hot water extract of galls are very effective for inflamed tonsils, while direct application of boiled and bruised galls on skin effectively cures any swelling or inflammation [8]. The constituents of its galls comprise mainly of hydrolysable tannins of tannic acid, gallic acid, ellagic acid and methyl gallate [9].

The aims of this study were to investigate the hepatoprotective potential of plant extracts containing mainly condensed tannins, as represented by areca seeds, and mainly hydrolysable tannins, represented by the galls of QI. Beneficial relationships between their antioxidant, anti-inflammatory and hepatoprotective potentials were evaluated by studying their antioxidant capacity in four different assays, their anti-inflammatory activity against the enzyme 5-lipoxygenase (5-LOX), and their hepatoprotective potential against liver injury induced by carbon tetrachloride (CCl_4_) in rats. Both extracts were also quantitatively analyzed by a reverse-phase HPLC and UV method to identify and determine the content of their phenolic principles.

## 2. Results and Discussion

**Figure 1:**
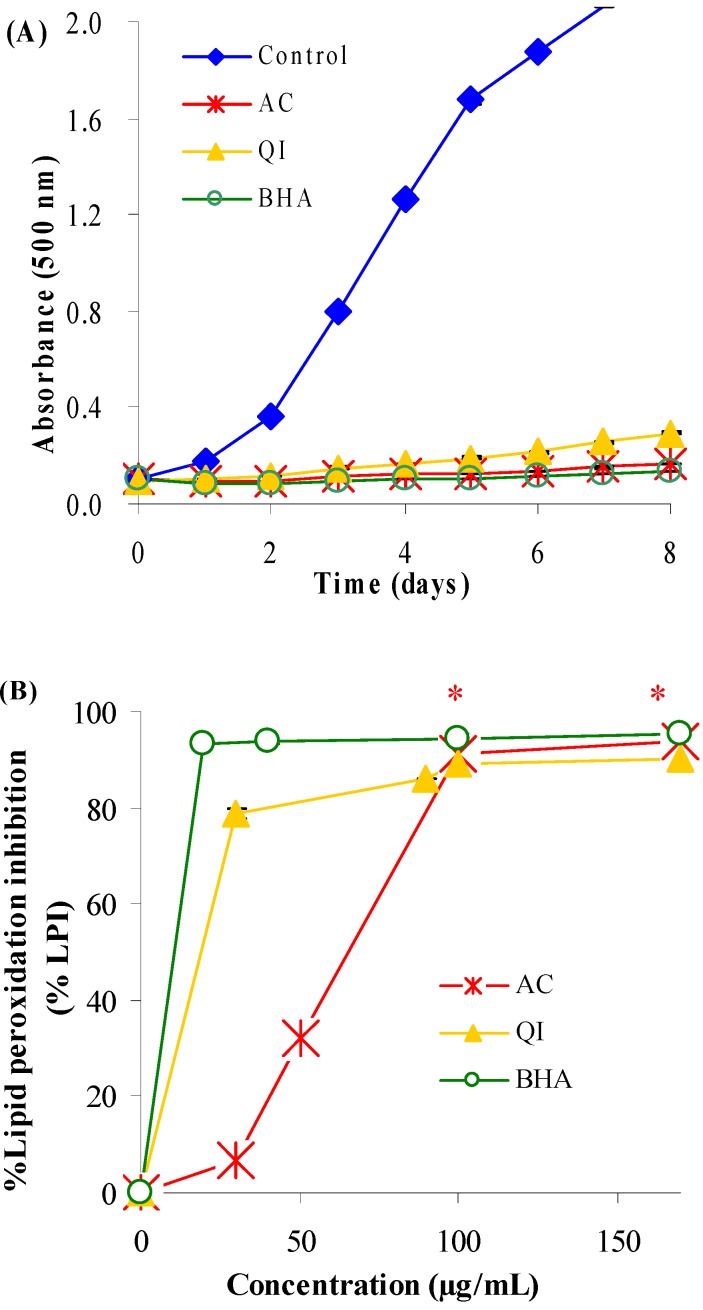
Effect of AC, QI and the positive reference (BHA) on lipid peroxidation of linoleic acid emulsion, as measured by formation of ferric thiocyanate. (A) Time course of linoleic acid peroxidation in the absence or presence of samples at 100 µg/mL concentration. (B) Percentage inhibition of lipid peroxidation as a function of sample concentration on day 8 of peroxidation. * No significantly different (p > 0.05) between percentage inhibition of BHA, AC and QI at concentration ≥ 100 µg/mL.

The results of all four types of *in vitro* antioxidant study were in good agreement, demonstrating that the aqueous extracts of AC and QI exhibit potent antioxidant activities. Figure 1A shows the time-course of lipid peroxidation in linoleic acid emulsion in the absence or presence of samples. All samples delayed oxidation of linoleic acid and exhibited higher activity than the control on the basis of low absorbance values. Figure 1B shows the percentage inhibition of lipid peroxidation on day 8 as a function of sample concentration. Lipid peroxidation was inhibited by AC and QI in a concentration-dependent manner. At the concentration of 30 µg/mL, percentage inhibition on day 8 decreased in the order: BHA (94%) > QI (79%) > AC (7%). However, at higher concentration (100 µg/mL), percentage inhibition of BHA (94%), QI (89%) and AC (91%) was not significantly different (*p* > 0.05). 

**Figure 2:**
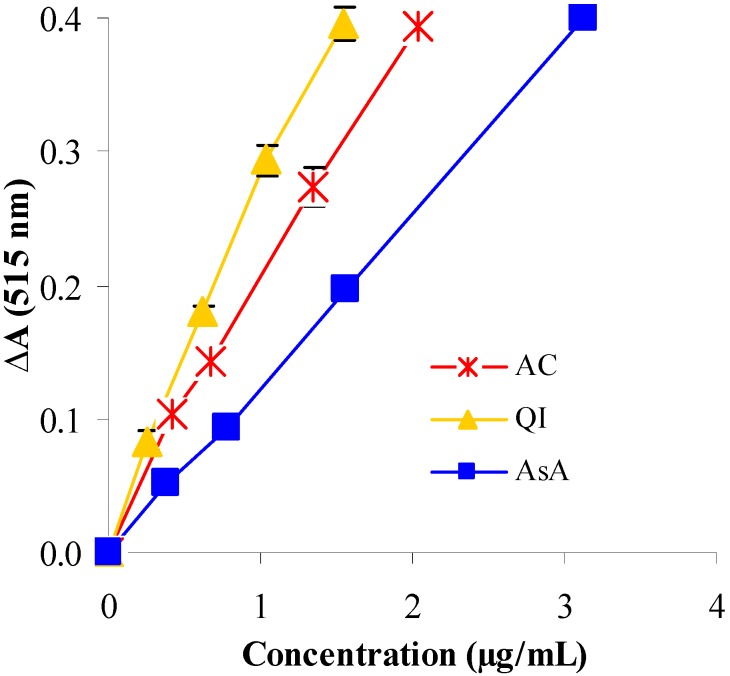
The reduction of DPPH radical measured by the decrease of absorbance at 515 nm, as a function of concentration of test samples and the reference antioxidant (AsA). The ΔA values, obtained after 30 min of reaction, are relative to those of the control excluding added samples or standard.

Reduction of DPPH by AC and QI occurred in a concentration−dependent manner (Figure 2). Reduction in the absorbance of DPPH was more pronounced in the presence of AC and QI compared with AsA, suggesting high free radical scavenging efficiency. The order of potency as judged from SC_50_ value was QI (0.98 µg/mL) > AC (1.38 µg/mL) > AsA (2.21 µg/mL) (Table 1). Scavenging of free radicals is one of the major antioxidant mechanisms that inhibit the chain reaction of lipid peroxidation. These results suggested that the extracts of AC and QI process free-radical scavenging activity which could be beneficial against pathological alterations caused by the free radical-generated lipid peroxidation.

**Table 1:** The concentration giving 50% inhibition of DPPH radical, hydroxyl radical, iron(II) chelating capacity and lipoxygenase activities of test samples and standard references. The values represent mean ± S.E.M. (n = 3).

Test samples	DPPH radical (SC_50_, µg/mL)	Hydroxyl radical (SC_50_, µg/mL)	Iron(II)-chelating (IC_50_, µg/mL)	Anti-lipoxygenase (IC_50_, µg/mL )
AC^a^	1.38 ± 0.08^b^	1,150.00 ± 0.00^ b^	2,966.67 ± 50.01^ b^	25.07 ± 0.23^ b^
QI^a^	0.98 ± 0.05^ b^	415.00 ± 15.00^ b^	6,100.00 ± 5.49^ b^	31.77 ± 0.54^ b^
Reference standards:				
Ascorbic acid (AsA)	2.21 ± 0.06	-	-	-
D-mannitol	-	8,150.00 ± 20.00	-	-
EDTA	-	-	5.35 ± 0.15	-
Nordihydroguaiaretic acid (NDGA)	-	-	-	59.50 ± 1.04

^a^ Concentration of AC and QI was calculated based on gallic acid equivalents (GAE); ^b^ Significantly different from the standard reference (*p* < 0.05).

**Figure 3:**
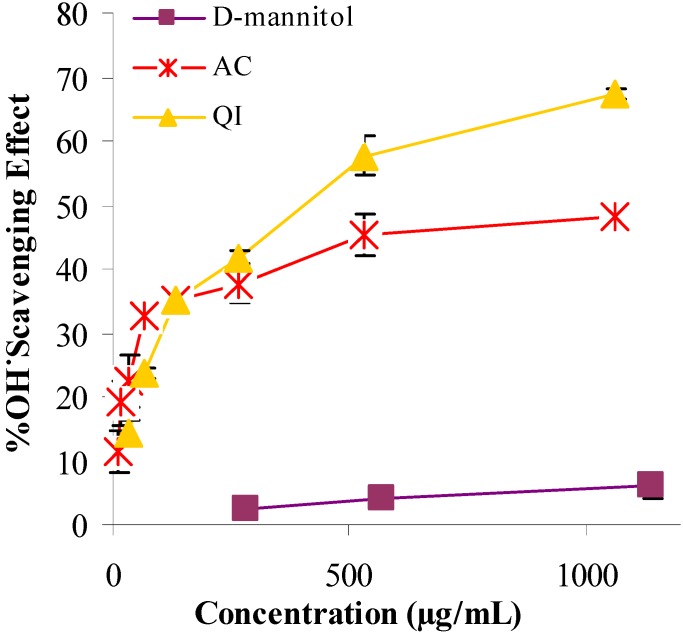
Hydroxyl radical scavenging activity of test samples (AC and QI) and the positive reference (D-mannitol) were spectrophotometrically measured at 532 nm using the deoxyribose assay.

Extracts of AC and QI demonstrated a remarkable capacity to scavenge hydroxyl radicals in the deoxyribose assay when compared with D−mannitol (Figure 3). The ability of AC, QI and D-Mannitol to scavenge hydroxyl free radicals was dependent on concentration. The scavenging potential was highest for QI, followed by AC and D−mannitol with SC_50_ values of 415, 1,150 and 8,150 µg/mL, respectively (Table 1). The extracts of AC and QI might therefore ameliorate oxidative stress−induced disorders, since hydroxyl radicals are generated under oxidative stress caused by various chemicals or disorders [10]. Both extracts also demonstrated a capacity for iron binding (Table 1), although their potency was much lower than EDTA. Based on the IC_50_ in Table 1, the metal-chelating effect decreased in the order: EDTA (5 µg/mL) > AC (2,967 µg/mL) > QI (6,100 µg/mL). This should also contribute to the inhibition of lipid peroxidation.

Extracts of AC and QI exhibited 5-LOX inhibitory activity, with IC_50_ values 25.07 and 31.77 µg/mL, respectively (Table 1). Values were two-fold less than the positive reference, NDGA (IC_50_ 59.50 µg/mL). This suggests the potent anti-inflammatory action of the extracts of AC and QI. The results support previous findings of [11] and [12] which demonstrated antioxidant and inhibitory activities of extracts of AC and QI against ear edema in mice. Inflammation plays a central role during induced acute hepatitis, and leukotrienes (the 5-LOX products of arachidonic acid metabolism) are extensively involved in inflammatory processes [13]. The inhibitory activities of AC and QI extracts against inflammation and 5-LOX could be a factor in the protective effect of these extracts against CCl_4_-induced hepatotoxicity in rats.

Since CCl_4_-induced liver injury is directly involved with the free radical and lipid peroxidation and it has been reported as the best characterized animal model of xenobiotic-induced free radical-mediated hepatotoxicity [14]; we, therefore, selected the CCl_4_-induced liver damage as a model in this study in order to reflect the antioxidant activities of both extracts and elucidate their hepatoprotective activity against CCl_4_. 

**Figure 4:**
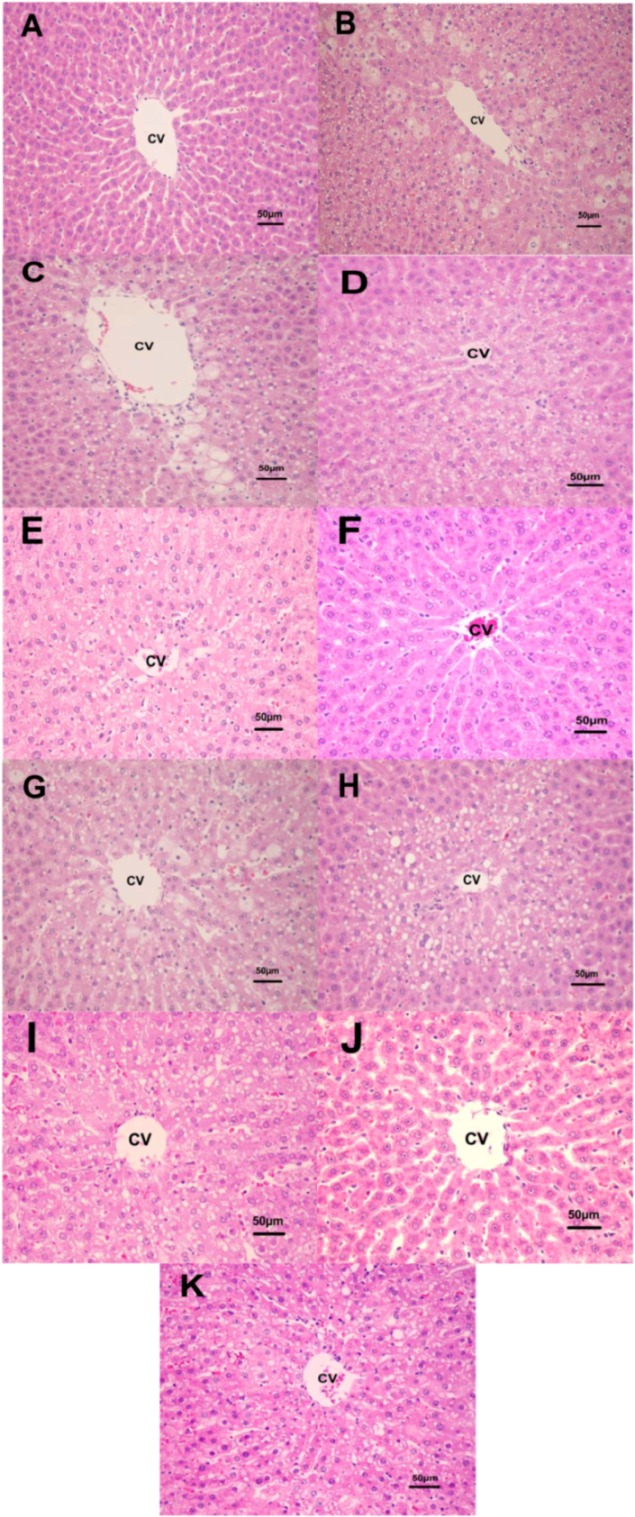
Histopathological changes of the liver (H and E, 200×) (A) normal control group, (B) CCl_4_ group, (C-E) extract treatment groups (CCl_4_ + AC 500, 1,000 and 2,000 mg/kg, respectively), (F) AC (2,000 mg/kg) alone, (G-I) extract treatment groups (CCl_4_ + QI 500, 1,000 and 2,000 mg/kg, respectively), (J) QI (2,000 mg/kg) alone and (K) CCl_4_ + silymarin (100 mg/kg) (CV, central vein).

Levels of marker enzymes of liver damage (ALT and AST) and biochemical parameter (MDA) are summarized in Table 2. Levels of ALT, AST and MDA increased significantly in the group treated with CCl_4_ compared with healthy control (*p* < 0.05). In contrast, the group treated with 500, 1000 and 2000 mg/kg of AC and QI significantly decreased (*p* < 0.05) the elevated levels of ALT, AST and MDA toward normalization in a dose-dependent manner. Although it seemed inferior to AC and QI (2000 mg/kg), the positive reference (silymarin) at 100 mg/kg also prevented elevation of serum enzymes and hepatic MDA. The percent protection of AC (67−85%) and QI (67−79%) at 2000 mg/kg was much higher than those of AC and QI at 500 and 1,000 mg/kg (18−33% and 37−50%, respectively) and silymarin (50−75%). There was no statistically significant different between the groups treated with AC, QI (2,000 mg/kg) and silymarin (*p* > 0.05). There were no changes in these parameters in the group treated with AC and QI (2,000 mg/kg) alone. Based on H&E stained tissue sections, administration of AC and QI (2,000 mg/kg) alone did not cause appreciable changes in liver histology throughout the study. CCl_4_ administration elicited extensive changes in liver morphology, including centrilobular vacuolar degeneration and necrosis. Pretreatment with AC and QI at 2,000 mg/kg preserved the hepatic architecture with few areas of vacuolar degeneration. These results clearly indicated the protection provided by AC and QI at 2000 mg/kg. In the liver cells of rats treated with 1,000 mg/kg of AC and QI and intoxicated with CCl_4_, the number of vacuolar degenerations was much more than the group treated with 2,000 mg/kg of AC and QI, but no necrosis could be seen. In the hepatic cells of rats treated with 500 mg/kg of AC and QI and intoxicated with CCl_4_, both vacuolar degeneration and necrosis were present, but less than the group intoxicated with CCl_4_ alone without any treatment (Figure 4). 

**Table 2:** Effect of AC and QI on serum ALT, AST and hepatic MDA in liver injury induced by CCl_4_ in rats. Values are mean ± SEM of five rats.

Groups	ALT	AST	MDA
	(U/L)	(U/L)	(nmol/g liver)
Normal	26.29 ± 2.31	60.40 ± 4.33	0.02 ± 0.00
CCl_4_	201.68^a^ ± 12.09	210.60^a^ ± 6.15	0.08^a^ ± 0.01
AC (500 mg/kg) + CCl_4_	170.61^b^ ± 4.38	176.91^b^ ± 8.34	0.06^b^ ± 0.00
	(17.71%)^c^	(22.43%)^c^	(33.33%) ^c^
AC (1,000 mg/kg) + CCl_4_	115.22^b^ ± 8.58	154.44^b^ ± 8.44	0.05^b^ ± 0.00
	(49.30%)^c^	(37.39%)^c^	(50.00%)^c,e^
AC (2,000 mg/kg) + CCl_4_	52.22^b^ ± 12.18	89.48^b^ ± 9.89	0.04^b^ ± 0.01
	(85.22%)^c,e^	(80.64%)^c,e^	(66.67%)^c,e^
AC (2,000 mg/kg)	26.67^d^ ± 6.62	80.83^d^ ± 12.71	0.03^d^ ± 0.00
QI (500 mg/kg) + CCl_4_	170.85^b^ ± 5.84	169.47^b^ ± 4.06	0.06^b^ ± 0.01
	(17.58%)^c^	(27.38%)^c^	(33.33%)^c^
QI (1,000 mg/kg) + CCl_4_	134.03^b^ ± 4.00	141.74^b^ ± 17.98	0.05^b^ ± 0.00
	(38.57%)^c^	(45.85%)^c^	(50.00%)^c,e^
QI (2,000 mg/kg) + CCl_4_	62.57^b^ ± 21.52	96.09^b^ ± 16.87	0.04^b^ ± 0.01
	(79.31%)^c,e^	(76.24%)^c,e^	(66.67%)^c,e^
QI (2,000 mg/kg)	29.87^d^ ± 7.70	84.14^d^ ± 19.10	0.03^d^ ± 0.00
Silymarin (100 mg/kg) + CCl_4_	80.78^b^ ± 34.52	98.57^b^ ± 24.71	0.05^b^ ± 0.01
	(68.93%)^c^	(74.59%)^c^	(50.00%)^c^

^a^ Significantly different from the control group (*p* < 0.05); ^b^ Significantly different from the group treated with CCl_4_ only (*p* < 0.05); ^c^ Percent protection in individual biochemical parameters (ALT, AST and MDA) from their elevated values caused by CCl_4_. The percent protection was calculated as 100 × (values of CCl_4_ control−values of sample)/(values of CCl_4_ control−values of control); ^d^ No significant difference compared with the control group (*p* > 0.05). ^e^ No significant difference compared with the positive reference (silymarin) group (*p* > 0.05).

It is seen from the results that CCl_4_ caused marked toxicity resulting in increased serum ALT (202 U/L), AST (211 U/L) and hepatic MDA (0.08 nmol/g liver). Treatment with polyphenolic extracts of AC and QI (2,000 mg/kg body weight) for 28 consecutive days produced a best protection of serum ALT (52−63 U/L), AST (89−96 U/L) and hepatic MDA (0.04 nmol/g liver) by 67−85% for AC and 67−79% for QI, respectively (Table 2). Administration of extracts of AC and QI appeared to facilitate the recovery from CCl_4_-induced hepatotoxicity. This phenomenon was also confirmed by the markedly reduced degree of histological liver damage compared with untreated rats (Figure 4). There was no appreciable change in the serum indices or liver damage in healthy rats treated with AC and QI alone, suggesting that the use of these extracts for potential hepatic protection after exposure to toxic substances would be safe. CCl_4_-induced hepatic damage is mediated by its free radical metabolites, which could readily react with macromolecules such as lipid and protein, leading to lipid peroxidation and cell injury [15]. This indicates the antioxidant and/or adaptive nature of the systems brought about by the extracts of AC and QI against the damaging effects of free radical produced by CCl_4_.

It was found from this study that AC contained mostly procyanidins (~80%) and QI contained mostly tannic acid (71%). Since procyanidins from plants have been reported to possess a broad spectrum of pharmacological activities including free radical scavenging, antioxidant, anti-inflammatory, anti-hepatotoxin and as well as being inhibitors of the enzymes phospholipase A_2_, cyclooxygenase and lipoxygenase [3,16,17]. Hydrolysable tannins such as tannic acid from plants have been reported to possess free radical scavenging activity, antioxidant activity, and to inhibit oxidative stress induced inflammation [18,19,20]. These results together with the present study implied that procyanidins, the major condensed tannins from areca seeds, and hydrolysable tannins from nutgalls of QI could be major contributors in the prevention of free radical mediated disorders including inflammation and hepatotoxicity. 

## 3. Experimental

### 3.1. Chemicals

2,2-Diphenyl-1-picrylhydrazyl (DPPH; ≥85%), gallic acid (GA; ≥98%), 2-thiobarbituric acid (TBA; ≥98%) and ascorbic acid (AsA; ≥99%) were purchased from Fluka (Buchs, Switzerland). (+)-Catechin hydrate (C; ≥98%), (−)-epicatechin (EC; ≥98%), Tannic acid (TA; ≥99%), 2-deoxy-D-ribose, 5-lipoxygenase (EC 1.13.11.12, 80,000 units/mg solid), D-mannitol (≥98%), ethylenediaminetetraacetic acid (EDTA; 99.8%), 3-(2-pyridyl)-5,6-bis(4-phenylsulfonic acid)-1,2,4-triazine (ferrozine; ≥99%), nordihydroguaiaretic acid (NDGA; ≥97%) and silymarin (batch 107K0762; 47% of silybin) were obtained from Sigma Chemical Co. (St. Louis, MO, USA). Butylated hydroxyanisole (BHA; 99.5%) was obtained from Nikki-Universal (Tokyo, Japan). Other chemicals and solvents used were of analytical grade. 

### 3.2. Animals

Male Wistar rats (150–200 g) were used for determination of the hepatoprotective effects of plant extracts on liver injury induced by CCl_4_. They were housed in well-ventilated room at 23 ± 2 °C, with a humidity of 65–70%, and a 12-h light/dark cycle. They were fed with a standard pellet diet with tap water *ad libitum*. Procedures involving laboratory animals were in accordance with the guidelines of the Mahidol University Animal Care and Use Committee (PY−ACUC).

### 3.3. Plant materials

Seeds of AC and nutgalls of QI were purchased from local market. Voucher specimens (RB 02035 and RB 02024) were deposited at the Museum of Natural Medicine, Faculty of Pharmaceutical Sciences, Chulalongkorn University, Thailand. Each plant material was macerated with 50% aqueous ethanol for three days. After solvent removal under reduced pressure, the extracts were defatted with hexane and evaporated to remove hexane and the aqueous residues, and lyophilized to yield 50.4 and 69.3% (w/w) of AC and QI extracts, respectively, [21]. Total phenolic content of each extract was analyzed by the Folin−Ciocalteu colorimetric method [22]. Extracts of AC and QI were found to contain 541.45 ± 4.30 and 824.27 ± 6.16 mg/g of total phenolic content expressed as gallic acid equivalent (GAE, mg/g of extract), respectively.

### 3.4. Standardization of plant extracts

The AC and QI extracts were analyzed to determine the contents of C, EC, GA and TA by using a reverse−phase high−performance liquid chromatography method. An isocratic mode was used and the mobile phase was phosphoric acid in water (pH 2)/acetonitrile (87.5:12.5). The flow rate was 1 mL/min and the injection volume was 20 μL. Detection was at 280 nm and identification of individual compound was based on a comparison of the retention time and UV spectrum of unknown peaks with those of reference authentic standards. The individual standards were quantified in the extracts, by comparison with the generated standard curves. QI extract was found to contain 8% of GA and 71% of TA whereas AC extract was found to contain 3.3% of C and 0.8% of EC. 

The AC extract was further analyzed for the quantitative determination of total amount of procyanidins. Oxidative depolymerisation of the extract was carried out as described by [23], using HCL in *n*-butanol (5:95), heated with reflux at 85 °C for 50 min. After depolymerisation, the sample was assayed for absorbance at 550 nm, which was used for the estimatation of the procyanidin content in the crude extract [24]. The AC extract gave high absorbance value at 550 nm after oxidative depolymerization and the total percentage content of procyanidins (with recalculation for cyanidine chloride) was determined as 79.6 ± 1.5%.

### 3.5. Assay of antioxidant activities

#### 3.5.1. Determination of inhibition of lipid peroxidation using the ferric thiocyanate (FTC) method

The FTC method [25] was used to determine *in vitro* inhibition of peroxidation of linoleic acid. Antioxidant activity was calculated as the percentage inhibition of peroxidation of linoleic acid versus control. BHA was used as the positive reference.

#### 3.5.2. Determination of radical scavenging activity using the DPPH test

The DPPH method [26] was used to determine the potential of scavenging of free radicals of each sample. Antiradical activity was calculated as the percentage of DPPH decoloration versus control. Results were expressed as the concentration of test samples that scavenged 50% of free radicals from the reaction mixture (SC_50_). AsA was used as the positive reference.

#### 3.5.3. Determination of hydroxyl free radicals scavenging activity

Damage to deoxyribose mediated by hydroxyl free radicals were determined from the formation of thiobarbituric acid-reactive substances (TBARS) measured spectrophotometrically at 532 nm [27]. D-Mannitol was used as the positive reference. Percentage inhibition was calculated against control. Results were expressed as the concentration of test samples that scavenged 50% of free radicals (SC_50_).

#### 3.5.4. Chelation of ferrous metal ions

The method of [28] was followed using EDTA as the positive reference. Percentage inhibition of the formation of ferrozine-Fe^2+^ complex was calculated and compared with that of control. Results were expressed as concentration of test samples that chelated 50% of metal ions (IC_50_).

### 3.6. Determination of in vitro anti−inflammatory activity by the inhibition of 5-LOX

The method of [29] was followed. Initial rate of reaction was determined from the slope of the linear portion of the curve. Percentage inhibition of enzyme activity was calculated by comparison with the positive control (NDGA). The extent of inhibition caused by test samples was expressed as the percentage of the compound necessary to achieve 50% inhibition (IC_50_).

### 3.7. Determination of the hepatoprotective effects of AC and QI on liver injury induced by CCl4 in rats

Rats were divided into eleven groups of five. Group 1 served as healthy control and received distilled water (1 mL/kg body weight per day, p.o.). Group 2 served as CCl_4_ treated control group and received distilled water (1 mL/kg body weight per day, p.o.). Group 3–5 (extract treatment) were treated with AC at 500, 1,000 and 2,000 mg/kg body weight per day. Group 6 (extract alone) was treated with AC 2,000 mg/kg body weight per day. Group 7–9 (extract treatment) were treated with QI at 500, 1,000 and 2,000 mg/kg body weight per day. Group 10 (extract alone) was treated with QI 2,000 mg/kg body weight per day. Group 11 received silymarin (positive reference) at 100 mg/kg body weight per day. All treatments with plant extracts and positive reference were given orally for 28 days. Except for group 1, 6 and 10, all the other groups received CCl_4_:corn oil (1:1, 0.25 mL/kg body weight, i.p.) on day 29. Twenty-four hours after CCl_4_ intoxication, rats were anaesthetized using diethyl ether, and fasting blood samples were collected from the abdominal aorta. Shortly afterwards, the animals were scarified by mild ether anaesthesia and the livers were immediately removed. Blood samples were allowed to clot at room temperature for 30 min and centrifuge at 1,000 × g for 10 min to obtain serum. The samples of serum and liver were stored at -80 ºC until analysis.

### 3.8. Analysis of biochemical parameters

#### 3.8.1. Assessment of liver functions

Liver damage was assessed by the estimation of serum activities of alanine aminotransferase (ALT) and aspartate aminotransferase (AST) using commercially available test kits from Audit (Ireland). Briefly, one hundred microliters of the sample serum were added to 1 mL of test reagent in 1 cm light path cuvette. After incubation at 37 °C for 1 min, measurement was made for the changes of optical density per minute (ΔOD/min) during the next 3 min at the wavelength of 340 nm. The enzyme activity was expressed as U/L and can be calculated as follows:

U/L = (ΔOD/min) × 1746

#### 3.8.2. Determination of lipid peroxidation

The mean malondialdehyde (MDA) content (nmol/g liver), a measure of lipid peroxidation, was assayed in the form of thiobarbituric acid-reacting substances (TBARS) [30].

### 3.10. Statistical analysis

Concentration of AC and QI was calculated based on GAE. Sample concentration providing 50% inhibition was calculated from the graph plotting inhibition percentage against sample concentration using SigmaPlot 2000 Demo (SPSS Inc., Chicago, IL, USA). All *in vitro* experiments were done in triplicate. Data are mean ± S.E.M. Statistical analyses were carried out using Minitab^®^ Release 14 for Windows (Minitab Inc., State College, PA, USA). Analysis of variance was done by ANOVA procedures. Significant differences between means were identified using Tukey’s pairwise comparison test at *p* < 0.05.

## 4. Conclusions

The reported information implied that procyanidins, the major condensed tannins from areca seeds, and hydrolysable tannins from nutgalls of QI could be major contributors in the prevention of free-radical-mediated disorders including inflammation and hepatotoxicity. These data should contribute to evidence-based traditional medicines for anti-inflammatory and hepatoprotective effects of both extracts.
